# SERPINA3 in glioblastoma and Alzheimer’s disease

**DOI:** 10.18632/aging.203603

**Published:** 2021-09-29

**Authors:** Emily S. Norton, Sandro Da Mesquita, Hugo Guerrero-Cazares

**Affiliations:** 1 Department of Neurosurgery, Mayo Clinic, Jacksonville, FL 32224, USA; 2 Neuroscience Graduate Program, Mayo Clinic Graduate School of Biomedical Sciences, Mayo Clinic, Jacksonville, FL 32224, USA; 3 Regenerative Sciences Training Program, Center for Regenerative Medicine, Mayo Clinic, Jacksonville, FL 32224, USA; 4 Department of Neuroscience, Mayo Clinic, Jacksonville, FL 32224, USA

**Keywords:** alpha-1 antichymotrypsin, glioblastoma, cerebrospinal fluid, aging, astrocyte, Alzheimer’s disease, SERPINA3

Glioblastoma (GBM) is the most common and aggressive primary brain tumor in adults. Although GBM can arise at any age, incidence increases with aging and the median age of diagnosis is 64 years. Aging-induced changes in gene expression and immune microenvironment contribute to the increase of GBM incidence and mortality in elderly patients [1]. An age-related factor that has been previously implicated in neurological aging and may contribute to increased glioma incidence is upregulation of SERPINA3. Increased SERPINA3 levels, glial-derived or otherwise, have been linked to altered brain function in aging and implicated in the development of brain amyloidosis in Alzheimer’s disease (AD). AD patients and animal models show a prominent cellular SERPINA3 expression in the vicinity of amyloid plaques and increased levels of extracellular SERPINA3 in direct contact with the amyloid beta (Aβ) aggregates [2]. Additionally, transgenic induction of SERPINA3 overexpression in astroglia resulted in increased brain Aβ burden presumptively by interfering with Aβ aggregation or degradation and clearance of amyloid aggregates [3]. Accordingly, constitutive loss of SERPINA3 led to a decrease in brain Aβ deposition in AD transgenic mice, an effect that was synergistically enhanced in mice lacking both SERPINA3 and apolipoprotein E [2,4]. Moreover, increased level of circulating SERPINA3 is correlated with the presence of brain microbleeds and white matter hyperintensities in elderly patients [5]. This indicates peripherally derived SERPINA3 is able to affect the brain directly, after crossing the blood brain barrier, or indirectly, by affecting the cellular response at border tissues like the meninges.

**Figure 1:**
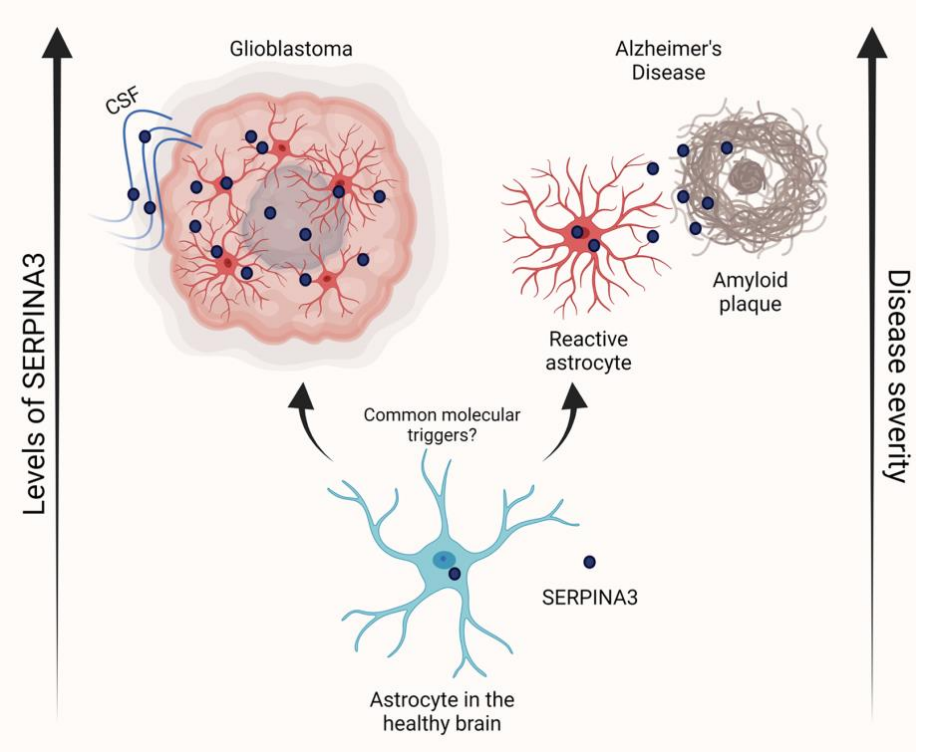
**SERPINA3 contribution to glioblastoma and Alzheimer’s disease**. Schematic depicting the contribution of rising SERPINA3 levels in the progression of age-related disorders glioblastoma and Alzheimer’s disease. Created in BioRender.com.

In the work by Lara-Velazquez et al. they describe an increased migratory behavior of GBM cells in the presence of cerebrospinal fluid (CSF) and identify SERPINA3 as a CSF-responsive factor *in vitro* [6]. Levels of SERPINA3 at the gene in protein level in patient samples correlate positively with glioma grade and negatively with patient survival. Additionally, SERPINA3 levels are increased in the CSF of high-grade glioma patients. Upon SERPINA3 knockdown, patient-derived GBM cells are unable to respond to CSF and have markedly decreased migration, clonal capacity, proliferation, colony formation, and CD133+ GBM stem-cell fraction, implicating the role of SERPINA3 in GBM malignant phenotypes. *In vivo*, silencing of the SERPINA3 gene reduces tumor formation capacity and intracranial tumor growth, resulting in increased median survival in rodents. Genetic rescue experiments indicate that increased GBM cell migratory behavior is dependent on endogenous SERPINA3 expression and cannot be rescued via the addition of recombinant SERPINA3. In sum, these results strongly implicate the neurological aging-associated factor SERPINA3 as an important promoter of glioma malignancy. The increased amount of SERPINA3 in the aging brain and its effects on brain tumors may contribute to the higher incidence of these malignancies in older patients (Figure 1).

The presence of brain tumors induces a progressive dysregulation of multiple homeostatic processes which results in altered angiogenesis, impaired immune response, reactive astrocytosis, and lymphatic drainage. Impaired glymphatic function, brain lymphatic drainage and meningeal immune response have been closely linked with accelerated cognitive decline and aggravated Aβ pathology in models of AD [7,8]. Future studies should investigate whether aging or increased Aβ impact the expression of SERPINA3 and other aging-associated factors at the brain meninges, namely within the dura or around leptomeningeal penetrating blood vessels. It would also be interesting to explore the importance of brain versus peripherally derived SERPINA3 and how these two sources affect brain function by promoting changes in CSF/interstitial fluid recirculation, gliosis and/or by modulating the immune response at the brain parenchyma and its meningeal borders.

Ultimately, Lara-Velazquez et al. present a compelling target in GBM that links the malignancy of these tumors with aging, immunomodulation, and CSF clearance. This information provides a basis for future studies examining the contribution of these factors to gliomagenesis and progression, and will likely lead to the development of novel therapeutics to target this deadly disease.

While GBM is considered a “cold tumor” due to its immunosuppressive nature, the aging brain is pro-inflammatory. However, in both situations there is an increase in the presence of reactive astrocytes and microglial cells with immune-suppressor activity. Both scenarios share the common presence of SERPINA3 as potential immunomodulator. The mechanisms responsible for regulating the balance of pro-inflammatory aging and anti-inflammatory tumor environment represent potential targets in the treatment of neoplasias and neurodegenerative diseases.
